# Object manufacture based on a memorized template: Goffin’s cockatoos attend to different model features

**DOI:** 10.1007/s10071-020-01435-7

**Published:** 2020-10-28

**Authors:** I. B. Laumer, S. A. Jelbert, A. H. Taylor, T. Rössler, A. M. I. Auersperg

**Affiliations:** 1 Department of Cognitive Biology, University of Vienna, Althanstr. 14, Vienna, 1090 Austria; 2 Department of Anthropology, University of California, 375 Portola Plaza, 341 Haines Hall, Box 951553, Los Angeles, CA 90095 USA; 3 School of Psychological Science, University of Bristol, Priory Road, Bristol, BS8 1TU UK; 4 School of Psychology, University of Auckland, Auckland, New Zealand; 5 Messerli Research Institute, University of Veterinary Medicine (other partner institutions: University of Vienna, Medical University of Vienna), Veterinärplatz 1, 1210 Vienna, Austria

**Keywords:** Emulation, Recall, Matching to sample, Mental imagery, Reverse engineering, Template matching, Tool manufacture

## Abstract

Although several nonhuman animals have the ability to recognize and match templates in computerized tasks, we know little about their ability to recall and then physically manufacture specific features of mental templates. Across three experiments, Goffin cockatoos (*Cacatua goffiniana*), a species that can use tools in captivity, were exposed to two pre-made template objects, varying in either colour, size (long or short) or shape (I or L-shaped), where only one template was rewarded. Birds were then given the opportunity to manufacture versions of these objects themselves. We found that all birds carved paper strips from the same colour material as the rewarded template, and half were also able to match the size of a template (long and short). This occurred despite the template being absent at test and birds being rewarded at random. However, we found no evidence that cockatoos could carve L-shaped pieces after learning that L-shaped templates were rewarded, though their manufactured strips were wider than in previous tests. Overall, our results show that Goffin cockatoos possess the ability to physically adjust at least the size dimension of manufactured objects relative to a mental template. This ability has previously only been shown in New Caledonian crows, where template matching was suggested as a potential mechanism allowing for the cumulative cultural transmission of tool designs. Our results show that within avian tool users, the ability to recreate a physical template from memory does not seem to be restricted to species that have cumulative tool cultures.

**Electronic supplementary material:**

The online version of this article (10.1007/s10071-020-01435-7) contains supplementary material, which is available to authorized users.

## Introduction

What shape are a fox´s ears? Most people visualize the corresponding image before answering the question (Pearson and Kosslyn 2015). Internal representations such as images can be stored as ‘templates’ in memory which can then be retrieved accordingly for visual pattern search and recognition (e.g. Kunda 2018). Humans can represent information in multiple ways and use mental templates to actively produce memorized patterns, for example, during mental imagery or mental simulation (Pearson and Kossyln 2015). In the Rey-Osterrieth complex figure test (Rey 1941), humans are asked to reproduce a sketch, first by copying it while it is present (referred to as ‘recognition’) and then after a short or longer delay by drawing it from memory (immediate and delayed recall). In non-human animals the ability to *recognize* information is typically assessed in delayed matching-to-sample tasks using computerized touchscreen designs, where the focus is usually on how long certain animal species can keep specific object features in memory (e.g. Truppa et al. 2014; White 1985; Goto and Wanabe 2009). *Recall*, on the other hand, requires the animal to specifically reproduce remembered information via a generative response (Basile 2018).

Notably, so far, we know very little about non-human animals’ ability to recall and physically reproduce specific object features from memory. In one neat example, using a computerized task similar to the Rey-Osterrieth complex figure test, rhesus monkeys reproduced simple shapes, after a delay, by aligning two or three coloured boxes on a touchscreen grid (Basile and Hampton 2011). Thus, we have evidence that monkeys can recollect specific shapes and then reproduce them by touching specific locations on a screen. More recently, an experiment with New Caledonian crows, a tool maker that is dependent on tool-obtained resources (Rutz et al. 2010), built on this by demonstrating that this species can actively make and modify an object’s properties to approximate a memorized template (Jelbert et al. 2018). Using a novel manufacture task, New Caledonian crows were presented with card rectangles of two different sizes and learnt that only one size (large: 40 × 60 mm or small: 15 × 25 mm), was rewarded. In the following test the birds were provided with very large sheets of card from which the birds could tear sections (i.e. manufacture their own card pieces) to insert into a vending machine to obtain rewards. During the test, the crows manufactured and inserted significantly larger card pieces when they had learnt that a large template was rewarded, compared to a small template. The results suggested that the birds were capable of flexibly recalling and reproducing at least one object property (small versus large size) in relative but not absolute proportions to a mental template (Jelbert et al. 2018).

These findings led Jelbert et al. (2018) to propose that having the capacity for mental template matching is a plausible account for the evidence of cumulative cultural evolution seen in this species. In the wild, New Caledonian crows are known to make at least three types of Pandanus tools which substantially differ in appearance depending on the geographic area that the birds inhabit (Hunt and Gray 2003). This regional variation has been argued to represent an example of cumulative cultural transmission (Hunt and Uomini 2016; Dean et al. 2014; St Clair et al. 2018; Hunt and Gray 2004). However, a plausible mechanism for the transmission of tool designs among birds was unclear, because New Caledonian crows show limited reliance on social learning in both captivity and the wild (Logan et al. 2016; Kenward et al. 2006; Holzhaider et al. 2010a, b). Jelbert et al. (2018) argued that, using mental template matching, juvenile birds could form a mental template of their parents’ tool designs through repeated exposure and use, and then reproduce these designs from memory. This ability could underpin the cultural transmission of tool designs in this species, and in turn supports the hypothesis that technical intelligence may be a driving force in the emergence of cumulative cultural evolution (Osiurak and Reynaud 2020; Taylor and Jelbert 2020). What is not yet clear is whether the abilities seen among New Caledonian crows are unique to this species, and whether a capacity for mental template matching developed as a consequence of New Caledonian crows’ cumulative tool culture. To date, only New Caledonian crows have been tested in a template matching task. Thus, it would be highly informative to understand whether other avian species, who do not demonstrate evidence of cumulative tool cultures in the wild, are similarly capable of manufacturing physical objects in accordance with a mental template. Here we aimed to test a highly capable avian tool maker which, unlike the New Caledonian crow (Rutz et al. 2010), does not appear to possess cumulatively transmitted tool cultures (O’Hara et al. 2018). Finding the ability to modify an object relative to a memorized template in another avian species would both further our understanding of recall abilities among non-human animals, and render it unlikely that this ability developed in one species of crow exclusively as an adaptation to a cumulative tool culture.

The Goffin’s cockatoo (*Cacatua goffiniana*) is an explorative and opportunist island parrot that has shown the capacity to solve a variety of tool-related problems in laboratory settings, equaling the performances of New Caledonian crows in many domains (Auersperg et al. 2012, 2014, 2016; 2017; Beinhauer et al. 2019; Habl and Auersperg 2017; Laumer et al. 2016, 2017). Notably, it can spontaneously innovate tool manufacture in the laboratory (Auersperg et al. 2012, 2014, 2016; Laumer et al. 2017; Osuna-Mascaró and Auersperg 2018), including the manufacture of the same object from different materials and different object types from the same material (Laumer et al. 2017). These birds can flexibly rip shapes from cardboard and paper, which makes a comparison with the crows feasible (Auersperg et al. 2016, 2017; Laumer et al. 2017). It is possible that the Goffin’s can also spontaneously innovate tool manufacture in wild settings (Osuna-Mascaró and Auersperg 2018) but they do not show tool cultures in their natural habitat, the Tanimbar archipelago (O’Hara et al. 2018).

To maintain comparability between species we used a similar paradigm as Jelbert et al. (2018) with slight modifications: As our birds are well habituated to being tested we did not use a vending machine to dispense rewards and as tool manufacture is somewhat constricted by the beak morphology of our study species (Auersperg et al. 2018) we used paper strips as templates rather than rectangular templates. Furthermore, whereas the previous setup only included two object properties (colour and size; Jelbert et al. 2018), we added an additional test condition (Shape test). The Colour test was designed to investigate whether the cockatoos would manufacture strips out of the same colour as previously rewarded templates. In the Size test, we tested whether the birds would manufacture short and long paper strips, depending on the previously rewarded template. To assess whether the Goffins were able to match the shape of an object, we trained them prior to the test to insert L-shaped paper objects over straight ones. Due to the Goffin cockatoos carving each strip via a large number of bite marks alongside the edge of the paper, to produce a L-shape subjects had to carve the object alongside the corner of the paper square.

## Methods

### Subjects, housing and experimental history

We tested five male and one female adult, captive-born and hand-reared Goffin’s cockatoos. All subjects participated in a Colour test and in Size tests I & II and four males participated in the Shape test (one subject lost motivation and one developed a sudden aversion to inserting paper). Subjects are permanently housed together in a large, enriched aviary with indoor and outdoor area (ca. 200 m^2^ ground space, up to 6 m high) at the Goffin Lab associated with the University of Veterinary Medicine (Vienna, Austria). All parrots are kept on ad libitum diet (boiled and raw seeds, fresh and dried fruits, boiled vegetables, fresh water) and participate in the experiments on a voluntary basis. As all experiments were appetitive, non-invasive and based exclusively on behavioural tests, they are not classified as animal experiments under the Austrian Animal Experiments Act (§ 2. Federal Law Gazette No. 501/1989). All animals had CITES certificates and were registered at the district’s administrative animal welfare bureau (Bezirkshauptmannschaft St. Pölten Schmiedgasse 4–6, A-3100; St. Pölten, Austria). These housing conditions comply with the Austrian Federal Act on the Protection of Animals (Animal Protection Act—§ 24 Abs. 1Z1 and 2;§25Abs.3—TSchG,BGBl.INr.118/2004Art.2).

Prior to this experiment all birds had participated in a study on social transmission of tool use and manufacture (only Figaro, Dolittle & Kiwi had sculpted stick-tools out of larch-wood, Pipin had used but not made tools; for details see Auersperg et al. 2014), four individuals were tested in a study on tool manufacture (only Figaro and Dolittle made stick-tools out of cardboard; for detailed results see Auersperg et al. 2016) and all subjects had participated in a hook-bending experiment (from the six subjects that were tested in present study only Fini manufactured hook tools and was consistently successful, Figaro was occasionally successful; see Laumer et al. 2017). For detailed information on the individual subjects see SI, section A, table S1.

### Apparatus and insertion training

**Fig. 1:**
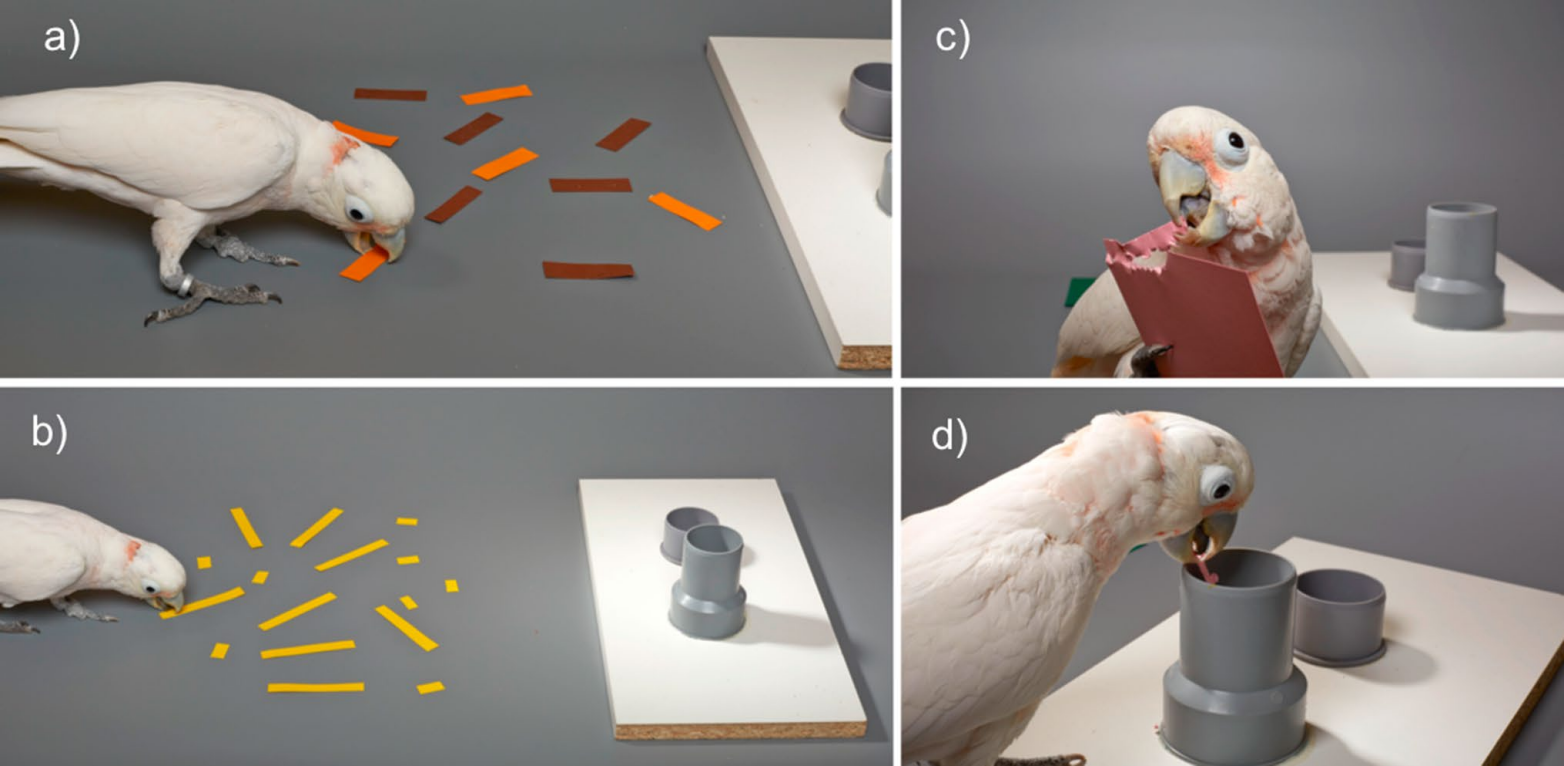
**a** Training in preparation for the Colour test. **b** Training in preparation for the Size test. **c** Test: Goffin carves a strip of paper by applying a large number of bite marks alongside the edge of the paper. Different colours of paper were used in each condition. **d** Test: Goffin inserts the manufactured strip into the large tube. Afterwards a food reward is placed in the small tube (Photos by Bene Croy)

Subjects were habituated to the apparatus. The apparatus consisted of a grey insertion tube (length 7 cm; diameter 6 cm) and a shorter tube used as a feeding tray, in which the experimenter placed the rewards (see Fig. 1). All birds had used cardboard strips as tools before (Auersperg et al. 2016; 2017). Note that in the present context the cockatoos were required to use the cardboard as a token rather than as a foraging tool. The Goffins were first trained to insert strips of white paper (5 cm in length) into the tube to receive a food reward (small piece of cashew). This was done by encouraging them to insert the strips (combination of tapping onto the tube with the fingertip; note that no secondary reinforcers other than food were used in later training phases). If they successfully inserted the strip into the tube in 30 consecutive trials they entered the card-ripping test (see below).

### Pretest: card-ripping test

Prior to the actual experiment, subjects were presented with a card-ripping test to examine whether they would spontaneously rip a strip of paper out of a white paper card and insert it. They first received three trials in which they had to insert a strip of white paper (5 cm; same length as in the insertion-training). Then the experimenter (IBL) placed a 10 × 10 cm piece of white paper card on the testing table. If the subjects successfully carved and dropped a strip into the tube, they were given a food reward (small piece of cashew). Subjects received a total of five more trials to move on to the next stage of the experiment (Colour test). If subjects did not manufacture a strip of paper, they received three insertion-trials and the card-ripping test trial was repeated on the subsequent testing day.

### Colour learning and colour test

Subjects first learned that only one colour out of two differently coloured paper strips was rewarded (Fig. 1a). The Goffins received a varying amount of trials per session depending on their individual motivation (usually between 10 and 30 trials), in which they had to choose between two differently coloured paper strips (5 cm in length). Subjects were sitting on the back of a chair while the experimenter placed the two items on the testing table. The side of the correct coloured strip was semi-randomly counterbalanced across sessions. During the entire testing period the experimenter wore mirrored sunglasses, avoided any head-movements and was not speaking to the animal. Directly after placing the strips, she signalled the bird to wait by extending her right arm with the palm facing towards the subject (wait-signal). After three seconds she removed her hand, thereby allowing the Goffin to leave its starting position. Only one colour was rewarded. If the Goffin chose the wrong colour, it was immediately placed for a duration of 30 s in a cage directly next to the testing table. Since the Goffin cockatoos are generally highly motivated to participate in the experiments, this timeout, although short in duration, served as a mild and effective treatment to show them their failure. Note that time-out was only used during training and never in any of the test conditions (Colour, Size and Shape test).

As soon as the birds were able to select the strip of the correct colour in at least ten consecutive trials, they received two discrimination test sessions per day, conducted directly after the other. In each session, eight strips of the correct colour and eight strips of the unrewarded colour were placed in a randomized fashion on the table. To eliminate stimulus-enhancement, the experimenter touched the 16 items all at once with spread hands before allowing the bird to leave its starting position. If the Goffin was able to select and drop all eight correct coloured strips in a row in both test sessions it passed the criterion and entered the Colour test, in which it had to manufacture a strip on its own. If not, it was tested again on the subsequent test day.

In the Colour test (see movie S1), in each trial two paper squares in the rewarded and unrewarded colour (10 × 10 cm in size) were placed simultaneously on the table. Only strips made from the correct colour were rewarded. Subjects received a total of two sessions of 12 trials each.

### Size learning and size tests I

The Goffins learnt that only strips of card of a specific size (either short or long) were rewarded (Fig. 1b). The paper that was used had a different colour than in the Colour test. We randomly divided the subjects in two groups, with one group being rewarded for inserting short strips, while the other group was rewarded for dropping long strips. Subjects received a varying number of trials per session depending on their individual motivation (usually between 10 and 30 trials), in which they had to choose between a short (2 cm in length) and a long piece of paper (8 cm in length). As in the Colour test, the subject was sitting on the chairback and the experimenter placed the two items on the table. Then the subject was allowed to leave its starting position. The side of the correctly sized strip was semi-randomly balanced across sessions. If the Goffin made the correct decision in the training it got immediately rewarded, if not it was placed in the cage for 30 s.

Before the Size test, the Goffins had to pass five error-free criterion-sessions in a row to be tested. In each of the sessions, eight short and eight long paper strips were placed in a randomized fashion on the testing table. Again, the experimenter touched the 16 items all at once to prevent stimulus-enhancement, before the subject was allowed to leave its starting position. If the cockatoo made a mistake, testing was continued on the subsequent testing day. Subjects received up to three sessions within one test day.

In the Size Test (see movie S1), subjects received 10 test trials per session. Before the first Size test trial and after every second Size test trial, subjects received a reminder phase, in which two short and two long strips were placed on the table. Only the previously learned size was rewarded. After correct insertion of the two reminder strips subjects received two Size test trials, in which the experimenter placed a 10 × 10 cm sized paper card on the table. Goffins were given the opportunity to manufacture a strip of paper from the large square and insert it into the tube. To prevent trial-and-error learning, the Goffins were rewarded at random on 50% of the Size test trials, regardless of the size of the ripped strip that they inserted. The Goffins received a total of two sessions of 10 Size test trials each.

### Reversal size learning and size test II

Once birds had completed their first Size test condition (long or short templates rewarded), they were tested again using the other size of template. Exactly the same size learning and Size test procedures were repeated, now rewarding the alternative size (long or short), using a new colour of card to draw the bird’s attention to the new task affordances.

### Shape test

The cockatoos were first trained to select L-shaped paper-objects (see SI, Figure S3; length of each side 4.3 cm, widths 1.4 cm) over straight ones (6 cm in length, widths 1.4 cm). They were rewarded for correct choices and received a time-out for negative choices only during the training. Subjects had to pass two out of three criterion-sessions error-free in a row (one session consisted of placing 16 items on the testing table: 8 L-shapes, 8 straight strips) to be tested in the Shape test.

In the Shape test (see movie S1), subjects received 10 test trials per session. Before the first Shape test trial and after every second Shape test trial, subjects first received a reminder phase, in which two L-shaped paper object and two straight ones were placed on the table. Only the L-shaped piece was rewarded. After correct insertion of the reminder strips, subjects received two Shape test trials, in which the experimenter placed a 10 × 10 cm sized paper card on the table.

Due to their manufacturing technique (each strip was carved by a large number of bite marks alongside the edge of the paper), producing a L-shaped object could only be achieved by carving the L-shape around the corner of the paper square. Subjects received a total of four sessions of 10 test trials each and were rewarded in only 50% of test trials, regardless of the shape of the carved strip.

Additionally, we investigated whether subjects could apply the previously learned shape-concept to another material (wire) and spontaneously form a L-shape out of a straight wire (without any additional wire-bending training; note that all subjects had participated in a hook-bending/unbending experiment using the same material; see Laumer et al. 2017). Subjects that previously were successful in selecting L-shaped paper objects, were now confronted with straight and L-shaped templates made out of wire. In the test the birds received a straight piece of wire (length 10 cm) and were tested in the exact same fashion as in the previous Shape test.

### Analysis

All trials were videotaped (JVC Camcorder) and coded in situ. All pieces of inserted and discarded pieces were collected and stored for measurement. In contrast to the New Caledonian crows, the Goffin cockatoos did not rip the paper pieces, but each strip was carved by a large number of bite marks alongside the edge of the paper until the bird cut in a curve after reaching a certain length. Therefore, the length of each strip was measured (Colour and Size test).

In the Shape test the widths of each bite mark along the ripped piece of the Shape test (40 pieces) and Size test (both Size tests combined = 40 pieces) were measured and compared (curved out ends under 1 cm were excluded from the analysis).

#### Analysis colour test

To evaluate choices in the Colour test we conducted binomial tests for individuals and used a Generalized Linear Mixed Model (GLMM; Baayen 2008) with binomial error structure and logit link function (McCullagh and Nelder 1989) to evaluate group performance. We included a random intercept of subject.

#### Analysis size test

To test whether the length of the carved-out pieces differed with the presented template size in the Size test we used a GLMM and controlled for group (short or long template first), session (1–4) and trial per condition (1–20). As length constitutes a continuous variable with a maximum of 10 cm (length of paper block) we used a beta distribution error structure and logit link function (Cribari-Neto and Zeileis 2010). Prior to fitting the model, we transformed length to be bound between 0 and 1 as recommended by Smithson and Verkuilen (2006). Additionally, we *z*-transformed trial and session to a mean of zero and a standard deviation of one to achieve easier interpretable estimates (Schielzeth 2010). We included a random intercept of subject to avoid pseudo-replication. Furthermore, the model entailed random slopes (Schielzeth and Forstmeier 2009; Barr et al. 2013) for session, trial and template size (manually dummy coded and centered) within subject. To account for daily differences of length produced per subject we included a random intercept combining subject and session (Sub.Sess) with a random slope of trial. As Jelbert et al. (2018) found an interaction of template size and number of trials we included this interaction term in the model. However, it did not have a significant effect and we therefore then excluded the interaction to obtain estimates for fixed effect. After fitting the model, we confirmed that there was no issue of collinearity (maximum Variance Inflation Factor: 1.176; assessed for model lacking the random intercept and slopes). Model stability was assessed by comparing estimates obtained from the model based on all data with estimates obtained from models in which the levels of random effects were excluded one at a time (Nieuwenhuis et al. 2012; function kindly provided by Roger Mundry). All estimates proved to be fairly stable (see Table S2 for model estimates). We first compared the full model with a null model lacking the main predictors (template size and trial) to avoid ‘cryptic multiple testing’ (Forstmeier and Schielzeth 2011) and then tested all predictors by single deletion, using likelihood ratio tests (Dobson 2002).

We further fitted the same model for each separate subject without the random intercept of subject (as there is only one subject per model) and without session (because of collinearity issues). Although we did not find a significant interaction of template size and trial in our model, to compare our results with the previous study on New Caledonian crows (Jelbert et al. 2018) we additionally fitted separate models for each template size. We included trial and group as fixed effect, random intercepts for subject and for subject and session combined (Sub.Sess) and random slopes of trial within both.

#### Analysis shape test

To test whether the shapes of the strips differed between conditions (short or long) we first determined maximum width of each strip and then compared them between conditions (Size test and Shape test) using a general linear model (i.e., assuming normally distributed and homogeneous residuals). To control for the possibility that individuals differed in the general widths of strips or that they responded differently to the two conditions, we further included individual and its interaction with condition into the model. Finally, we also included session and trial number to control for potential learning or fatigue. As an overall test of the effect of condition (i.e., as a full null mode comparison; Forstmeier and Schielzeth 2011), we compared this full model with a null model lacking condition and its interaction with individual.

To check for normality and homogeneity of the residuals we inspected a qq-plot of the residuals and residuals plotted against fitted values (Quinn and Keough 2002; Field 2005), which did not reveal strong deviations from these assumptions. We estimated model stability by means of DFBeta (Field 2005), which revealed the model to be stable. Collinearity was no issue (maximum Variance Inflation Factor: 1.333; assessed for model lacking the interaction.

**Fig. 2:**
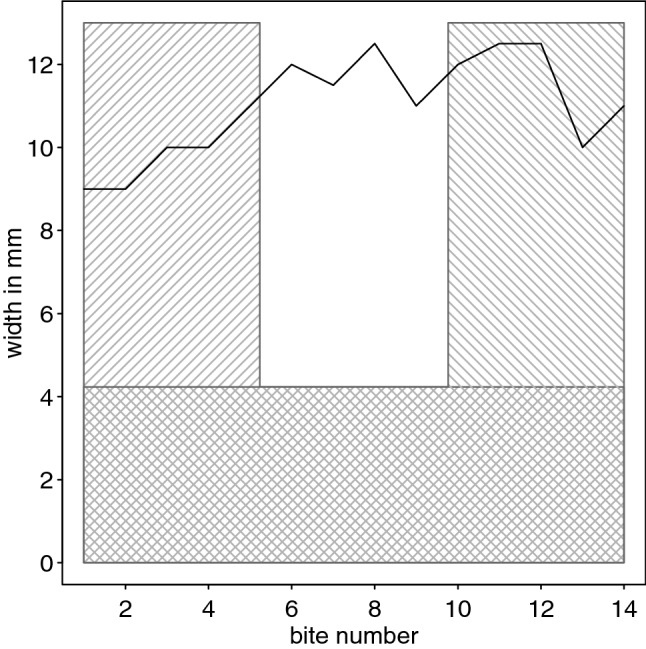
Illustration of the method used to estimate the degree match of the shape produced by the individual (black line) and the template (L-shaped grey polygons). Since the potential wider end of the shape produced could be on either end of it, we used two version of the L-shaped template, one with the vertical part on its left and one with the vertical part on the right side (vertical polygons with diagonal patterns). The horizontal bases therefore overlap and are illustrated with both diagonal patterns. Furthermore, we aligned the length of the base of the L-shaped template such that it matched the length of the shape the animal produced (*x*-axis)

To test whether the shapes the individuals produced resembled the templates we correlated their widths with the relative width of the template. We used a relative measure as subjects might have produced a similar shape but smaller in size. To do this we first measured the length of each piece and calculated the width relative to it (according to the original template). We then aligned the length of the template with the length of each piece produced (after aligning their bases). Note that when carving the strips the cockatoos placed a large number of bite marks alongside the edge of the paper, sometimes producing variations in widths. Since a potential bump in the width of the shapes the individuals produced could be on either end of them, we correlated them with template once in each of its two possible orientation and chose the larger of the resulting correlation coefficients (Fig. 2). We used Spearman's rank correlation coefficient as an estimator of the degree of similarity between the template and the shape the animals produced. We used the L-shaped template for shapes produced in the shape test as well as those produced in the size test condition. If the Goffins indeed matched the shapes they produced to the L-shape provided in the shape test condition, we would expect the correlation coefficients to be higher in this condition as compared to the size test condition.

All statistical tests were conducted in R (R Core Team 2018) (versions 3.6.1 and 3.5.1; RStudio (RStudio Team 2016) version 1.1.453). We used the packages ‘car’ (Fox and Weisberg 2019) (version 3.0-3) to assess collinearity (function vif) and ‘glmmTMB’ (Brooks et al. 2017) (version 0.2.3) to fit the mixed model (function glmmTMB). Further we used the function lm to fit the general linear model and lmer to fit the binominal model. Plots were drawn with ‘ggplot2’ (Wickham 2016) (version 3.2.1.) and base R.

## Results

### Colour test

In the Colour test all subjects selected the correctly coloured paper card above chance expectation to carve the strips (binomial significance cut-off: 18 out of 24; correct choices: Figaro and Kiwi = 24, Pipin and Konrad 23, Fini 22 and Dolittle 20 out of 24). This also held true for group comparison with an estimated probability to choose the correct colour of 0.954 (GLMM: estimate = 3.023, SE ± 0.551, *z* = 5.48, *p* < 0.001). Five out of the six birds carved the paper strip in the correct color in their very first trial. Subject Fini discarded an already manufactured strip of the wrong colour and then carved one out of the correctly coloured paper square and inserted it. The length of the strips varied within individuals throughout the course of the experiment.

### Size tests

**Fig. 3:**
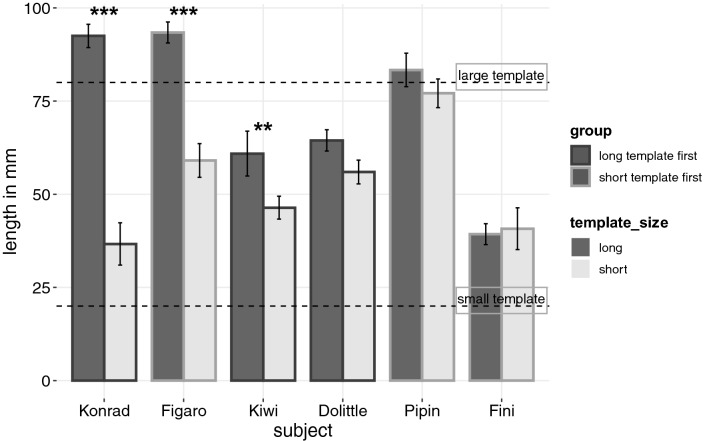
Mean length and standard error (black vertical lines) of the carved strips in the Size test for each subject (dark grey/left bars: Size test with long templates; light grey/right bars: Size test with short templates). Horizontal dashed lines indicate the length of the short (20 mm) and long (80 mm) templates. Colour of outline of each bar: dark grey outline = long template first, light grey outline = short template first. GLMMs: *p < 0.05, **p < 0.01, ***p < 0.001

**Fig. 4:**
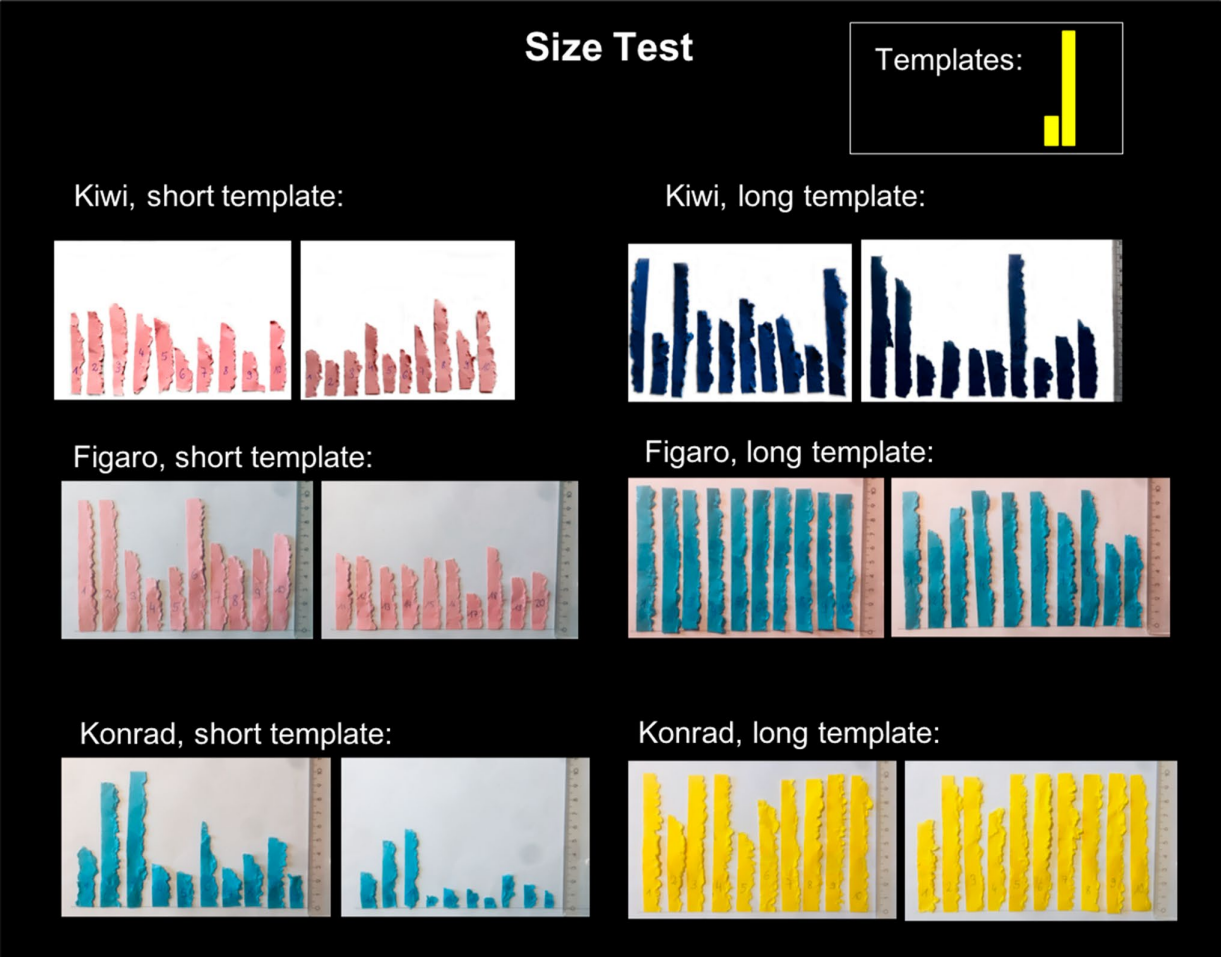
Manufactured strips of the Size Test of the three subjects that carved differently sized strips depending on previously experienced short or long templates (GLMMs; Kiwi: *p* < 0.01, Figaro & Konrad: *p* < 0.001)

In the Size tests the Goffin cockatoos manufactured shorter pieces when they had previously learned that short templates were rewarded and longer strips when long templates were previously rewarded (mean_short template_ = 52.7 ± 23.6 mm; mean_long template_ = 72.3 ± 25.7 mm). We found a combined effect of template size, trial and their interaction (GLMM: full-null model comparison: *χ*^2^ = 7.8, *df* = 3, *p* = 0.05). However, when tested by single deletion the interaction term was not significant (GLMM: estimate − 0.27, SE ± 0.19, *χ*^2^ = 1.89, *p* = 0.169). The reduced model (lacking the interaction term) showed a trend towards shorter pieces being made when the template was short (estimate = − 1.049, SE ± 0.489, *χ*^2^ = 3.310, *p* = 0.069). None of the other predictors (group, session or trial) were significant (see Table S2 for model output). On an individual level three of the six subjects carved differently sized strips depending on the size of the respective template (GLMMs: *p* < 0.01; see model output SI Table S3 and Figs. 3, 4).

When inspecting the conditions (long or short template) separately we found that birds produced shorter pieces as the number of trials increased in sessions with the short template (GLMM: estimate = − 0.436, SE ± 0.197, *χ*^2^ = 4.292, *df* = 1, *p* = 0.038). Interestingly, the group starting with the short template made longer pieces in this condition than birds with short templates in later sessions (estimate = 0.971, SE ± 0.366, *χ*^2^ = 5.126, *df* = 1, *p* = 0.024). No effect of trial or group was found in sessions with the long template (full-null (lacking trial and group) model: *χ*^2^ = 0.26, *df* = 2, *p* = 0.878; see model output Table S4).

### Shape test

To investigate whether subjects would match the shape of a template we conducted a Shape test. All birds previously learned that a L-shaped paper object was rewarded whereas a straight paper strip was not rewarded. Four individuals passed the criterion to be tested in the Shape test. At test, two individuals showed a difference in their strip manufacture behaviour and end products compared to the Size tests (Konrad and Kiwi, see `*individual strip manufacture`* below). Their data were therefore analyzed.

**Fig. 5:**
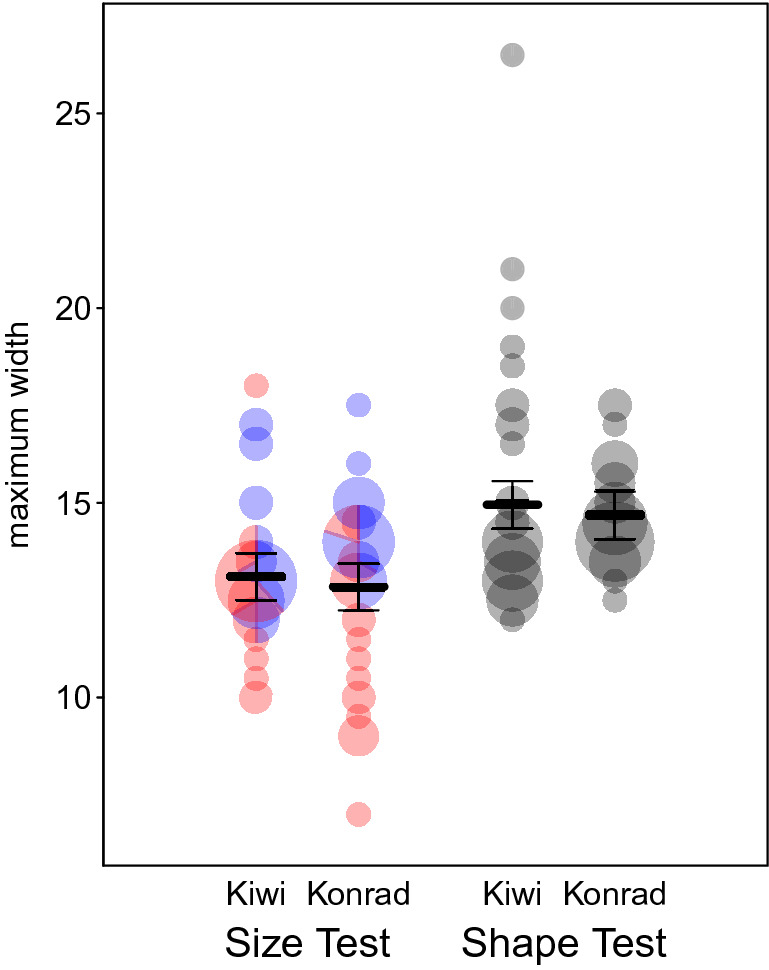
Maximum width (in mm) of strips in the two conditions and separately for the two individuals. The area of the circles corresponds to the number strips per individual, condition, and width (range: 1–12). In the size test condition two sizes were used. The proportions of red and blue of each dot depict tests with shorter (red) and longer (blue) template strips

Overall, the full-null model comparison revealed a clearly significant result (*F*(2, 154) = 11.159, *p* < 0.001). As the interaction between individual and condition did not reveal significance (estimate = 0.062, SE =  ± 0.674, *t*(154) = 0.093, *p* = 0.926; SI, Table S5) we removed it from the model. This reduced model revealed a clearly significant effect of condition whereby strips were clearly wider than in the Size test (SI, Table S6; Fig. 5). To rule out confounding of the comparison between conditions with the fact that in the Size test we used strips of two different lengths (short template = 2 cm, long template = 8 cm) we fitted an additional model in which we coded the factor condition with three levels (short, long, and shape). This revealed strips in the shape condition to be significantly wider than those in the short (estimate = − 2.887, SE ± 0.426, *t*(154) = − 6.771, *p* < 0.001) and marginally non-significantly wider than those in the long condition (estimate = − 0.788, SE ± 0.426, *t*(154) = − 1.847, *p* = 0.067).

However, the similarity between the manufactured shapes and the L-shaped template provided in the Shape test was not obviously larger in the Shape test as compared to the Size test (see SI, Figure S3).

### Individual strip manufacture observations in the shape test

**Fig. 6:**
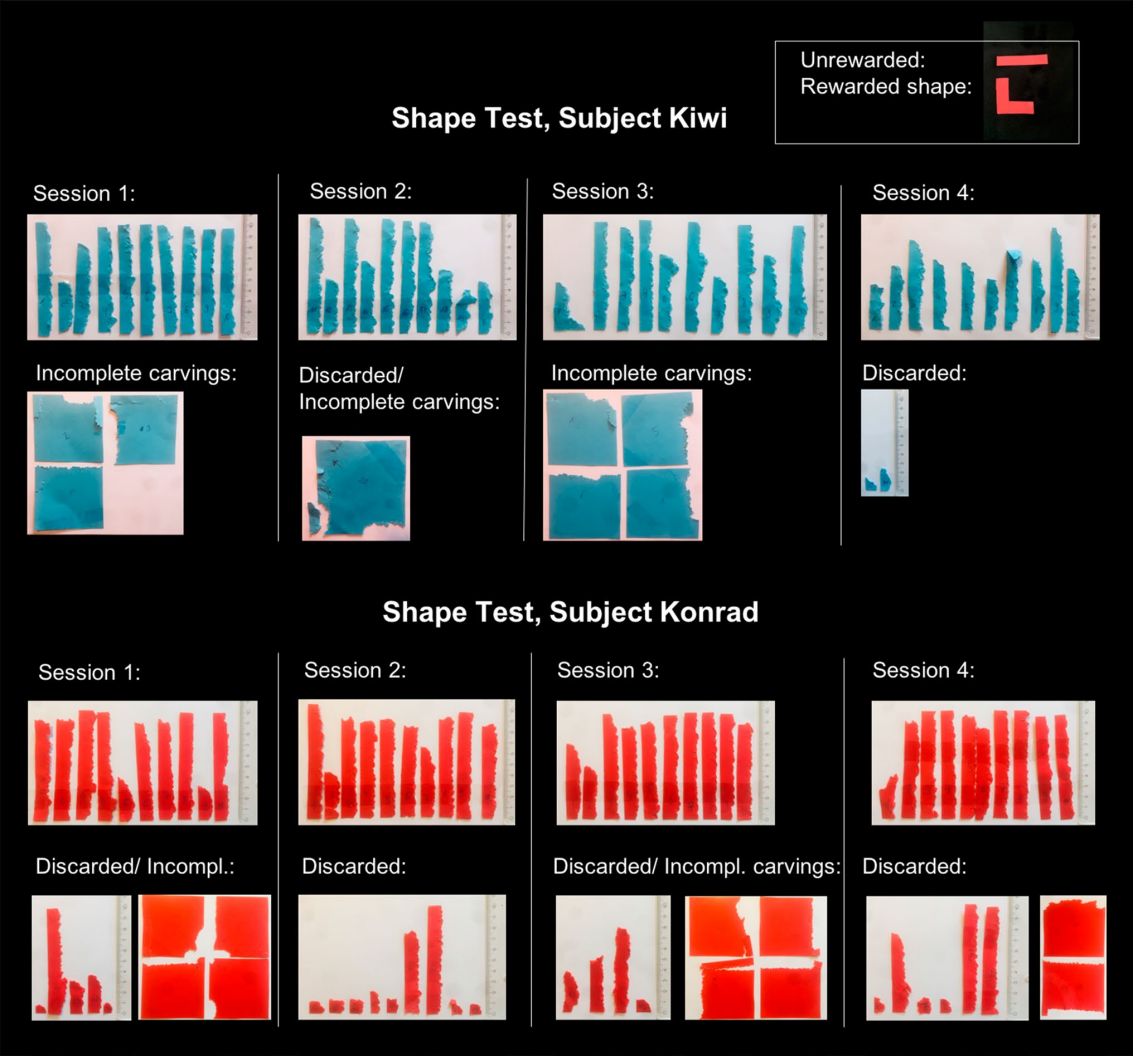
Manufactured strips and discarded/incomplete carvings of Kiwi and Konrad in the Shape Test

Compared to the previous Size test, Kiwi and Konrad showed clear differences in their manufacturing process in the Shape test. Overall, Konrad discarded a total of 24 manufactured pieces during the 40 test trials of the Shape test, compared to a total of 12 discarded pieces with both Size tests combined (40 trials, see SI, Figure S1). Ten of the discarded objects in the Shape test were carved by cutting off one corner of the original paper square, whereby he folded one of these items at one end (see Fig. 6, session 2). Occasionally Konrad carved out up to two pieces only partially before ripping of the final piece (trial 5, 6, 7, 21, 25, 26, 30). In Trial 31 he manufactured a piece that differed in shape when compared to the other strips (Fig. 6; please note that he manufactured and discarded a paper strip that was carved around the corner in the previous Size test as well (short template, S1).

In his second Shape test trial Kiwi started a total of three times to incompletely carve out a paper object until he finally ripped out the final piece and inserted it. This happened as well in Trial 3, 5, 20, 21 and 25 (between two and three incomplete carvings per trial; total number of trials = 40). In trial 19, Kiwi folded over the end of the carved paper strip prior to insertion and in trial 37 he folded one end of the carved paper strip in a 90**°** angle before inserting it (see Fig. 6). In trial 21, he carved a paper strip closely resembling the L-shaped template in absolute length (please note that he manufactured a similar strip in the previous Size test as well (short template, Trial 9).

### Shape test with bendable material

Kiwi was the only subject that was immediately able to apply the previously learned shape-concept to another material (wire) and was therefore trained with an L-shaped wire as template (see SI, Figure S2). Nevertheless, it took him 11 sessions to reach the criterion to be without mistakes for two sessions. In the test though, Kiwi never tried to bend the wire but instead immediately inserted the straight wire-piece.

## Discussion

Actively matching specific features of an object could be classified as an ill-structured problem, as the end product is known, but no information on how it is achieved is given (Cutting et al. 2014; Rutz et al. 2018). Here we found that Goffin’s cockatoos, similar to tool-specialized NC crows, spontaneously manufactured paper strips that matched the colour and, on an individual level, the size (short vs. long) of previously experienced, pre-made templates. An additional L-shape template test revealed no evidence for a respective shape approximation, although subjects manufactured wider pieces compared to the Size test and showed different behaviour during their manufacture process. Our results suggest that Goffin’s cockatoos can recall and achieve the active assimilation of an object to specific features such as size and colour of a memorized object through manufacture, despite being rewarded at random and in the absence of the respective template.

When given the choice between two differently coloured paper squares, similar to the New Caledonian crows (Jelbert et al. 2018), all Goffin’s cockatoos spontaneously manufactured strips of the same colour as the previously rewarded template. Five of the six subjects selected the correct color to manufacture objects from the first trial onwards. Surprisingly, in a more abstract touch screen setup the same bird had difficulties with a simple matching-to-sample setup including different colours (Gruber 2016). Several animal species, including birds, have previously been shown improve performance in matching-to-sample tasks when using physical rather than virtual objects (e.g. Wright and Delius 1994; Spetch and Friedman 2006; Stephan et al. 2014; O’Hara et al. 2015).

All four adult and two of the four subadult New Caledonian crows manufactured larger objects when previously rewarded for large templates than when rewarded for small templates (Jelbert et al. 2018). This was similarly the case for three out of the six cockatoos on an individual level, and showed as a trend on the group level. However, in most individuals, this behaviour was less prominently expressed than in the New Caledonian crow. The most accurate New Caledonian crow in the size test modified already detached pieces to reduce their size before inserting them in the vending machine, thereby providing tentative evidence for an attempt to match not just the relative, but the absolute size of the previously rewarded templates. Here, the most accurate Goffin, the adult male Konrad, made paper strips that were roughly similar to the short and long templates not only in relative but also in absolute length. The findings of the size test supplement previous results showing how Goffin’s cockatoos adjust the lengths of straight cardboard strips relative to the varying (yet visible) distance of the of an out-of-reach food reward (Auersperg et al. 2018) while saving effort in manufacture.

In the Shape test the cockatoos faced morphological and ergonomic constraints (distance between the beak tip and the edge of the horizontal part of the upper mandible allows only to carve out objects in a certain width; for pictures see Auersperg et al. 2018). Moreover, due to the carving technique used by the cockatoos (each strip was carved by a large number of bite marks alongside the edge of the paper), producing a L-shaped object could only be achieved when carving the shape alongside one corner of the paper square. Kiwi´s carved strip in Trial 21 proves that this task is not impossible for the cockatoos to carry out. However, he did this only once and manufactured a similar strip in the previous Size test as well (condition: short template, Trial 9). Statistically we looked only at the data for two birds in the shape test (Kiwi and Konrad) as they both applied different manufacturing techniques in the shape test compared to the Size test. Although the products of these two cockatoos were indeed significantly wider than in the Size tests, the manufactured pieces in the Shape test were statistically not more ‘bumpy’ and thus more similar to a L-shaped template than compared to the Size test. Interestingly, Konrad discarded a larger number of manufactured items before inserting the final object into the target tube compared to his behavior in the size tests and Kiwi frequently incompletely carved out two to three paper objects until manufacturing the final one. Konrad showed other alterations in his manufacture process including carving out objects by cutting off the corner of the original paper square and once produced a shape that completely differed in shape compared to all previously manufactured objects. Furthermore, both cockatoos bent some manufactured objects in a 90**°**degree angle before insertion (Konrad once, Kiwi twice; this behaviour was never shown by any of the cockatoos in the Size tests). The resulting products of these trials would achieve the original L-shaped template in a three-dimensional modality. However, the only cockatoo that reached the criterion for being tested with a bendable wire in Shape test 2, never bent the wire in the test condition. This was, however, not overtly surprising as some of our birds showed problems manipulating wire in the past and the respective subject previously failed to bend a wire in a tool-related context (Laumer et al. 2017).

Long-term captivity seems to affect the performance in cognitive tasks in some species (reviewed in Cheng and Byrne 2018). While Jelbert et al. (2018) tested wild-caught birds in their study, we tested captive reared birds in Austria. Nevertheless, our previous research suggests that rearing history does not have a strong impact on performance when it comes to technical problem solving in Goffin’s cockatoos: In a recent test battery of 20 artificial tasks, motivated wild-caught birds solved a similar number of problems at a similar rate as laboratory-raised birds (Rössler et al. 2020). Thus, it is unlikely that long-term captivity affects the performance in physical problem-solving tasks in our test species.

In summary, similar to a previous study in New Caledonian crows (Jelbert et al. 2018), we found that individual animals were able to recall and assimilate two (length and colour) features of a template. Like in the crows, the products made by the birds in the Size test should be regarded as assimilations based on the memorized models and not as absolute matches of the latter (with exceptions; see above). Finding the ability to physically produce object features of a template in a parrot that has the capacity for tool innovation (Auersperg et al. 2012; Osuna-Mascaró and Auersperg 2018, Laumer et al. 2017) but, so far, seems to lack a dependency on tool use and does not have cumulative tool cultures (O’Hara et al. 2018; Mioduszewska et al. 2019) suggests that the latter is not a necessary condition in order for this ability to evolve. We suggest it is likely that crows and parrots may share other inherent properties that facilitate such skills.

A possible origin of the ability for physical template matching (Jelbert et al. 2018) could be vocal template matching abilities, which are required for song learning (Slater 1983). Song birds, parrots and hummingbirds have the capacity for hearing and then reproducing complex auditory input: juvenile vocal learners listen to song templates from conspecifics and then continuously match their own vocalization to the vocal template (Slater 1983,1986; Marler 1970). Notably, and different to many songbirds, the capacity of parrots to match sounds towards a specific goal held in memory, often requires the use of various body movements, specifically those involving tongue and beak (e.g. Patterson and Pepperberg 1994; Warren et al. 1996), is not restricted to a sensitive developmental phase but is maintained in both sexes throughout adulthood (Pepperberg 1994; Pepperberg et al. 1991; Hile et al. 2000; Bradbury and Balsby 2016). Cockatoos, like most parrots (though not all), are open-ended learners that can imitate various sounds, so the likelihood of their having the same kind of template for vocal learning as songbirds is unlikely. However, similar to songbirds they have systems of cerebral vocal nuclei for controlling memorized vocalizations. Neurological research suggests that these nuclei are linked and even descend from brain areas involved various limb and body movements (Feenders et al. 2008). It is thus possible that the ability to match physical object properties is linked to sound matching/vocal learning as suggested by Jelbert et al. (2018). However, it is likely that parrots and corvids use a different kind of template for vocal learning than for physical template matching. Nevertheless, without further research this has to remain somewhat speculative at this point.

To investigate subjects’ ability to recall and reproduce object properties from memory into further depth, a promising next step would be to use either a similar task design or a touchscreen design (similar to Basile and Hampton 2011), so requiring subjects to switch flexibly between templates and to reproduce them after increasing delays.

## Electronic supplementary material

Below is the link to the electronic supplementary material.

Supplementary file1 (MP4 340558 kb)

Supplementary file2 (XLSX 109 kb)

Supplementary file3 (DOCX 1393 kb)

## Data Availability

The dataset supporting this article has been uploaded as part of the electronic supplementary material.

## References

[CR1] Auersperg AMI, Szabo B, von Bayern AMP, Kacelnik A (2012). Spontaneous innovation in tool manufacture and use in a Goffin’s cockatoo. Curr Biol.

[CR2] Auersperg AMI, von Bayern AMI, Weber S, Szabadvari A, Bugnyar T, Kacelnik A (2014). Social transmission of tool use and tool manufacture in Goffin cockatoos (*Cacatua goffini*). Proc R Soc B.

[CR3] Auersperg AMI, Borasinski S, Laumer I, Kacelnik A (2016). Goffin’s cockatoos make the same tool type from different materials. Biol Lett.

[CR5] Auersperg AMI, Köck C, Pledermann A, O'Hara M, Huber L (2017). Safekeeping of tools in Goffin's cockatoos, *Cacatua goffiniana*. Anim Behav.

[CR4] Auersperg AMI, Köck C, O’Hara M, Huber L (2018). Tool making cockatoos adjust the lengths but not the widths of their tools to function. PLoS ONE.

[CR6] Baayen RH (2008). Analyzing linguistic data.

[CR7] Barr DJ, Levy R, Scheepers C, Tily HJ (2013). Random effects structure for confirmatory hypothesis testing: keep it maximal. J Memory Lang.

[CR8] Basile BM, Vonk J, Shackelford T (2018). Recall. Encyclopedia of animal cognition and behavior.

[CR9] Basile BM, Hampton RR (2011). Monkeys recall and reproduce simple shapes from memory. Curr Biol.

[CR10] Beinhauer I, Bugnyar T, Auersperg AMI (2019). Prospective but not retrospective tool selection in the Goffin’s cockatoos (*Cacatua goffiniana*). Behaviour.

[CR11] Bradbury JW, Balsby TJ (2016). The functions of vocal learning in parrots. Behav Ecol Sociobiol.

[CR12] Brooks ME, Kristensen K, van Benthem KJ, Magnusson A, Berg CW, Nielsen A, Skaug HJ, Maechler M, Bolker BM (2017). glmmTMB balances speed and flexibility among packages for zero-inflated generalized linear mixed modeling. R J.

[CR13] Cheng K, Byrne RW (2018). Why human environments enhance animal capacities to use objects: evidence from keas (*Nestor notabilis*) and apes (*Gorilla gorilla, Pan paniscus, Pongo abelii, Pongo pygmaeus*). J Comp Psychol.

[CR14] Cribari-Neto F, Zeileis A (2010). Beta regression in R. J Stat Softw.

[CR15] Cutting N, Apperly IA, Chappell J, Beck SR (2014). The puzzling difficulty of tool innovation: why can’t children piece their knowledge together?. J Exp Child Psychol.

[CR16] Dean LG, Vale GL, Laland KN, Flynn E, Kendal RL (2014). Human cumulative culture: a comparative perspective. Biol Rev.

[CR17] Dobson AJ (2002). An introduction to generalized linear models.

[CR18] Feenders G, Liedvogel M, Rivas M, Zapka M, Horita H, Hara E (2008). Molecular mapping of movement-associated areas in the avian brain: a motor theory for vocal learning origin. PLoS ONE.

[CR19] Field A (2005). Discovering statistics using SPSS.

[CR20] Forstmeier W, Schielzeth H (2011). Cryptic multiple hypotheses testing in linear models: overestimated effect sizes and the winner’s curse. Behav Ecol Sociobiol.

[CR21] Fox J, Weisberg S (2019). An R companion to applied regression.

[CR22] Goto K, Watanabe S (2009). Visual working memory in jungle crows (*Corvus macrorhynchos*) in operant delayed matching-to-sample. Japanese Psychol Res.

[CR23] Gruber R (2016) Comparing matching and non-matching abilities in Goffin cockatoos (*Cacatua goffiniana*). Master’s Thesis, University of Vienna, Vienna

[CR24] Habl C, Auersperg AMI (2017). The keybox: Shape-frame fitting during tool use in Goffin’s cockatoos (*Cacatua goffiniana*). PLoS ONE.

[CR25] Hile AG, Plummer TK, Striedter GF (2000). Male vocal imitation produces call convergence during pair bonding in budgerigars *Melopsittacus undulatus*. Anim Behav.

[CR26] Holzhaider JC, Hunt GR, Gray RD (2010). Social learning in New Caledonian crows. Learn Behav.

[CR27] Holzhaider JC, Hunt GR, Gray RD (2010). The development of pandanus tool manufacture in wild New Caledonian crows. Behaviour.

[CR28] Hunt GR, Gray RD (2003). Diversification and cumulative evolution in New Caledonian crow tool manufacture. Proc R Soc B Biol Sci.

[CR29] Hunt GR, Gray RD (2004). Direct observations of pandanus-tool manufacture and use by a New Caledonian crow (*Corvus moneduloides*). Anim Cogn.

[CR30] Hunt GR, Uomini N (2016). A complex adaptive system may be essential for cumulative modifications in tool design. Jpn J Anim Psychol.

[CR31] Jelbert SA, Hosking RJ, Taylor AH, Gray RD (2018). Mental template matching is a potential cultural transmission mechanism for New Caledonian crow tool manufacturing traditions. Sci Rep.

[CR32] Kenward B, Rutz C, Weir AAS, Kacelnik A (2006). Development of tool use in New Caledonian crows: inherited action patterns and social influences. Anim Behav.

[CR33] Kunda M (2018). Visual mental imagery: a view from artificial intelligence, *Cortex*, 105. ISSN.

[CR34] Laumer IB, Bugnyar T, Auersperg AMI (2016). Flexible decision-making relative to reward quality and tool functionality in Goffin cockatoos (*Cacatua goffiniana*). Sci Rep.

[CR35] Laumer IB, Bugnyar T, Reber SA, Auersperg AMI (2017). Can hook-bending be let off the hook? Bending/unbending of pliant tools by cockatoos. Proc R Soc B.

[CR36] Logan CJ, Breen AJ, Taylor AH (2016). How New Caledonian crows solve novel foraging problems and what it means for cumulative culture. Learn Behav.

[CR37] Marler P (1970). Birdsong and speech development: Could there be parallels? There may be basic rules governing vocal learning to which many species conform, including man. Am Sci.

[CR38] McCullagh P, Nelder JA (1989). Generalized linear models.

[CR39] Mioduszewska B, O’Hara M, Haryoko T, Auersperg A, Huber L, Prawiradilaga DM (2019). Notes on ecology of wild Goffin’s cockatoo in the late dry season with emphasis on feeding ecology. Treubia.

[CR40] Nieuwenhuis R, te Grotenhuis M, Pelzer B (2012). influence.me: tools for detecting influential data in mixed effect models. R J.

[CR41] O’Hara M, Huber L, Gajdon GK (2015). The advantage of objects over images in discrimination and reversal learning by kea, *Nestor notabilis*. Anim Behav.

[CR42] O'Hara M, Mioduszewska B, Haryoko T, Prawiradilaga DM, Huber L, Auersperg A (2018). Extraction without tooling around—the first comprehensive description of the foraging- and socio-ecology of wild Goffin´s cockatoos (*Cacatua goffiniana*). Behaviour.

[CR43] Osiurak F, Reynaud E (2020). The elephant in the room: what matters cognitively in cumulative technological culture. Behav Brain Sci.

[CR44] Osuna-Mascaró AJ, Auersperg AMI (2018). On the brink of tool use? Could object combinations during foraging in a feral Goffin's cockatoo (*Cacatua goffiniana*) result in tool innovations?. Anim Behav Cogn.

[CR45] Patterson DK, Pepperberg IM (1994). A comparative study of human and parrot phonation: acoustic and articulatory correlates of vowels. J Acoust Soc Am.

[CR46] Pearson J, Kosslyn SM (2015). The heterogeneity of mental representation: ending the imagery debate. PNAS.

[CR47] Pepperberg IM (1994). Vocal learning in grey parrots (*Psittacus erithacus*): effects of social interaction, reference, and context. Auk.

[CR48] Pepperberg IM, Brese KJ, Harris BJ (1991). Solitary sound play during acquisition of English vocalizations by an African Grey parrot (*Psittacus erithacus*): possible parallels with children´s monologue speech. Appl Psycholinguist.

[CR49] Quinn GP, Keough MJ (2002). Experimental designs and data analysis for biologists.

[CR50] R Core Team (2018) R: A language and environment for statistical computing. R Foundation for Statistical Computing

[CR51] Rey A (1941). L’examen psychologique dans les cas d’encephalopathie traumatique. Archives de Psychologie.

[CR52] Rössler T, Mioduszewska B, O’Hare M, Huber L, Prawiradilaga DM, Auersperg AMI (2020). Using an innovation arena to compare wild-caught and laboratory Goffin´s cockatoos. Sci Rep.

[CR53] RStudio Team (2016) RStudio: integrated development for R. RStudio, Inc.

[CR54] Rutz C, Bluff LA, Reed N, Troscianko J, Newton J, Inger R, Kacelnik A, Bearhop S (2010). The ecological significance of tool use in New Caledonian crows. Science.

[CR55] Rutz C, Hunt GR, St Clair JH (2018). Corvid technologies: how do new caledonian crows get their tool designs?. Curr Bio.

[CR56] Schielzeth H (2010). Simple means to improve the interpretability of regression coefficients. Methods Ecol Evol.

[CR57] Schielzeth H, Forstmeier W (2009). Conclusions beyond support: overconfident estimates in mixed models. Behav Ecol.

[CR58] Slater PJB (1983). Bird song learning: theme and variations. Perspect Ornithol.

[CR59] Slater PJB (1986). The cultural transmission of bird song. Trends Ecol Evol.

[CR60] Smithson M, Verkuilen J (2006). A better lemon squeezer? Maximum-likelihood regression with beta-distributed dependent variables. Psychol Methods.

[CR61] Spetch ML, Friedman A (2006). Comparative cognition of object recognition. Comp Cogn Behav Rev.

[CR62] St Clair JJH, Klump BC, Sugasawa S, Higgott CG, Colegrave N, Rutz C (2018). Hook innovation boosts foraging efficiency in tool-using crows. Nat Ecol Evol.

[CR63] Stephan C, Steurer MM, Aust U (2014). Discrimination of holograms and real objects by pigeons (*Columba livia*) and humans (*Homo sapiens*). J Comp Psychol.

[CR64] Taylor A, Jelbert S (2020). The crow in the room: New Caledonian crows offer insight into the necessary and sufficient conditions for cumulative cultural evolution. Behav Brain Sci.

[CR65] Truppa V, De Simone DA, Mortari EP, De Lillo C (2014). Effects of brief time delays on matching-to-sample abilities in capuchin monkeys (*Sapajus spp*.). Behav Brain Res.

[CR66] Warren DK, Patterson DK, Pepperberg IM (1996). Mechanism of American English vowel production in a grey parrot (*Psittacus erithacus*). Auk.

[CR67] White KG (1985). Characteristics of forgetting functions in delayed matching-to-sample. J Exp Anal Behav.

[CR68] Wickham H (2016) ggplot2: elegant graphics for data analysis. R package version 3.2.1. Springer

[CR69] Wright AA, Delius JD (1994). Scratch and match: pigeons learn matching and oddity with gravel stimuli. J Exp Psychol Anim Behav Process.

