# Supplementary material for: Object manufacture based on a memorized template: Goffin’s cockatoos attend to different model features
Source: Anim Cogn. 2020 Oct 28;24(3):457–70. doi: 10.1007/s10071-020-01435-7 (PMC8128754; doi:10.1007/s10071-020-01435-7)
Supplement: Supplementary file 3 — Supplementary file3 (DOCX 1393 kb) [file 10071_2020_1435_MOESM3_ESM.docx]

**Object manufacture based on a memorized template: Goffin’s cockatoos attend to different model features**

AUTHORS: Laumer, I. B., Jelbert S.A., Taylor A.H., Rössler T., Auersperg, A.M.I.

**Supplementary Material**

1. Subject information

**Table S1** Names, sex and age of the 6 Goffin cockatoos.

| Name | Sex | Hatched |
| --- | --- | --- |
| **Figaro** | male | 2007 |
| **Pipin** | male | 2008 |
| **Kiwi** | male | 2010 |
| **Konrad** | male | 2010 |
| **Dolittle** | male | 2011 |
| **Fini** | female | 2007 |

1. Training

Before testing started only two subjects had previously made cardboard tools, we therefore trained four more birds to increase our sample. Two of the subjects, Figaro and Dolittle, functioned as card-ripping demonstrators for the remaining four subjects, since they already had experience in carving strips out of cardboard in order to use them as stick-tools (Auersperg et al., 2016).

*Training of naïve subjects*

After being allowed to retrieve food from the apparatus using a ready-made tool, the four subjects previously naïve in cardboard tool manufacture (Fini, Pipin, Kiwi & Konrad) were offered a cardboard block (15 x 6 cm) in front of the baited apparatus from the previous study (Auersperg et al., 2016). If subjects successfully made cardboard tools they received up to nine additional trials within the same session (with the same procedure). If they failed to do so within 10 min of the same trial they received another session on the next testing day. If a bird failed to manufacture a cardboard tool within five sessions, a demonstrator bird (Figaro or Dolittle) made and used three tools in succession while the observer waited in an adjacent parrot cage. Each naïve bird was thereafter given the opportunity again to make cardboard tools themselves (once again for 10 minutes) for a maximum of five demo-followed sessions. Subjects that still failed to make cardboard tools were presented with cardboard sheets that were perforated along the edge of the material (with perforations alongside the longer edge of the cardboard, in a 1 cm interval) prior to being retested with the original material (until they became continuously successful).

Fini was successful for a single time in session five (before demos or pre-punched sheets). She thereafter received demonstrations. After succeeding to make a functional tool each for a single time after demo sessions one and two she became continuously successful (10 consecutive trials) after demo session three.

Kiwi, Pipin and Konrad did not succeed before receiving pre-punched cardboard sheets. All made functional tools from regular cardboard sheets from session one after experiencing pre-punched sheets, Kiwi became continuously successful at doing so from session three (S1: 1 tool; S2: 1 tools; S3: 10 tools), Pipin from session 6 ( S1: 2 tools; S2: 1 tool; S3: 1 tool; S4: 3 tools; S5: 3 tools; S6:10 tools) and Konrad from session 7 ( S1: 1 tool; S2: 1 tool; S3: 1 tool; S4: 1 tool; S5: 1 tool, S6: 4 tools; S7: 10 tools).

*Reminder trials for cardboard-tool-making subjects*

Subjects that already had experience with cardboard tool manufacture were given 2 sessions of reminder trials (1 session consisted of 10 trials) to test whether they still made tools out of cardboard after a testing pause of several months (Auersperg et al., 2016). All subjects were continuously successful.

1. Pictures of discarded strips in Size test


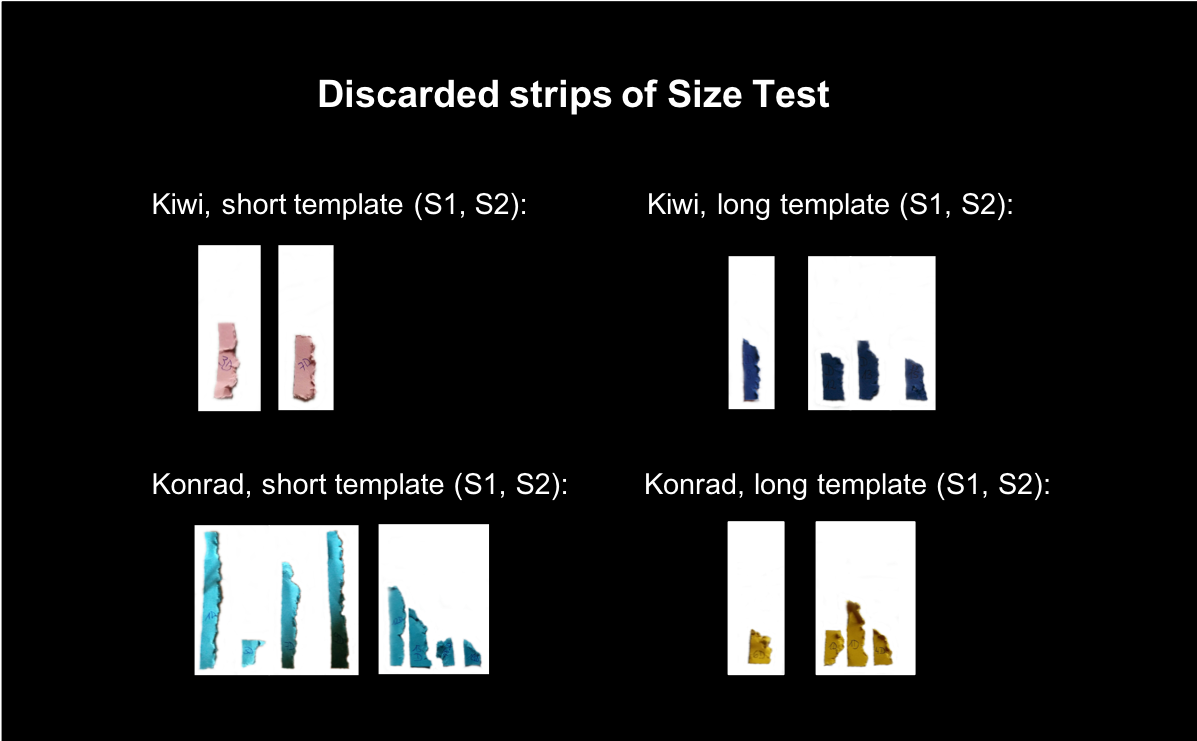


**Figure S1** Picture of the discarded strips of Kiwi and Konrad in the Size test.

1. Picture of the material used in the Shape test with bendable material


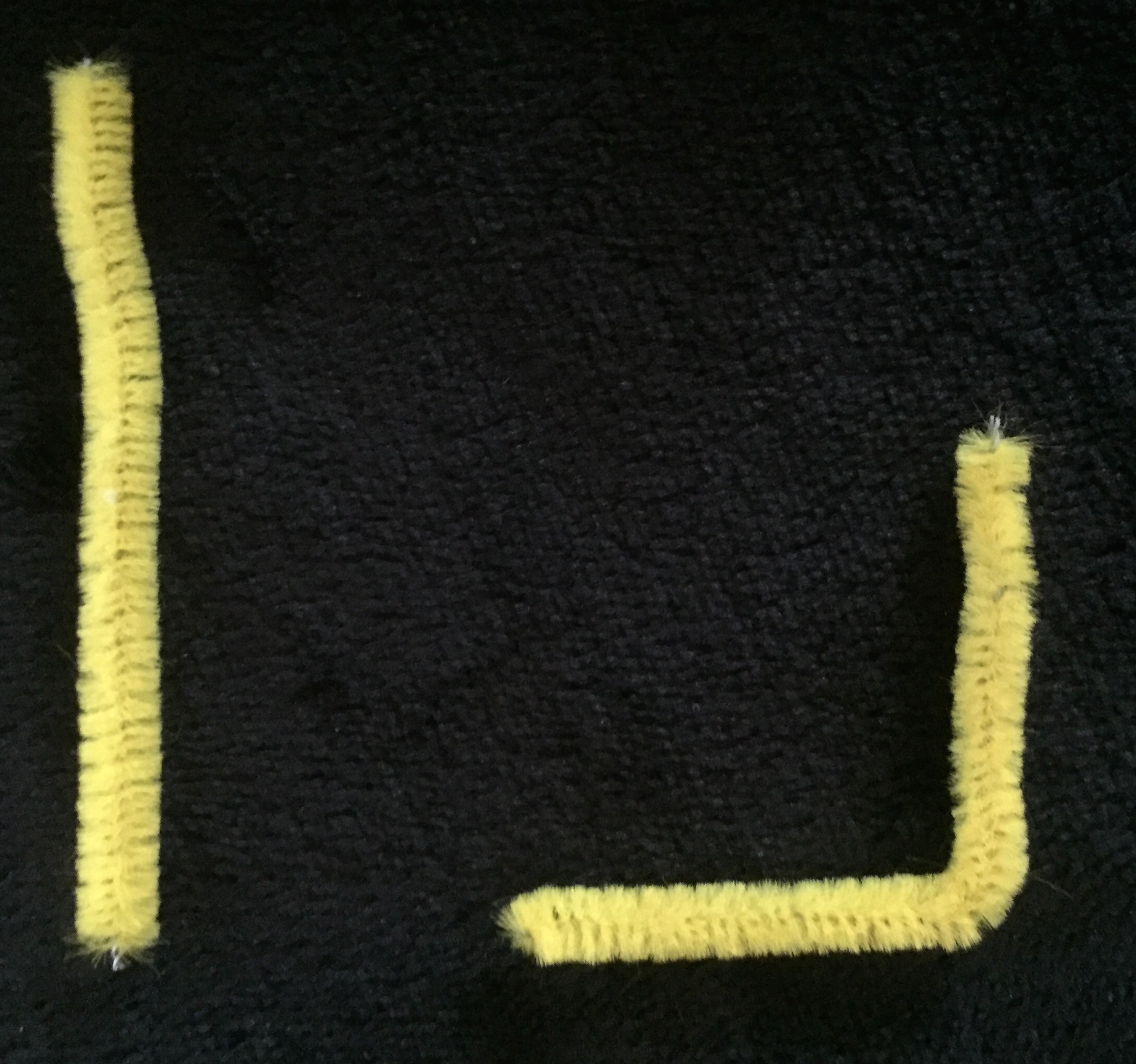


**Figure S2** Picture of the straight and L-shaped template made out of wire (pipecleaner).

1. Supplementary results

**Figure S3** Similarity (correlation coefficient) between the shapes the animals produced and the L-shaped template, separately for the two individuals in the Shape test and the Size test. Bold horizontal lines indicate median values, boxes span for the first to third quartiles and whiskers illustrate quantiles (2.5 and 97.5%). Crosses depict minimum and maximum values.

**Table S2** Results of the reduced model (lacking interaction term) for length (Size test; estimates, together with standard errors, and confidence intervals, as well minimum and maximum of estimates obtained when excluding data points one at a time).

| **term** | **estimate** | **SE** | **lower CI** | **upper CI** | **𝜒^2^** | **df** | **P** | **min** | **max** |
| --- | --- | --- | --- | --- | --- | --- | --- | --- | --- |
| intercept | 1.146 | 0.422 | 0.329 | 1.954 | ^(1)^ | ^(1)^ | ^(1)^ | 0.737 | 1.344 |
| template size ^(2)^ | -1.049 | 0.489 | -2.027 | -0.066 | 3.310 | 1 | 0.069 | -1.443 | -0.664 |
| trial ^(3)^ | -0.199 | 0.131 | -0.450 | 0.062 | 2.295 | 1 | 0.130 | -0.25 | -0.065 |
| group ^(4)^ | 0.452 | 0.484 | -0.521 | 1.328 | 0.813 | 1 | 0.367 | 0.127 | 1.014 |
| session ^(3)^ | -0.002 | 0.213 | -0.452 | 0.406 | 0.000 | 1 | 0.993 | -0.139 | 0.097 |

^(1)^ not indicated because of having a very limited interpretation

^(2)^ dummy coded and centered with template size ‘large’ being the reference category

^(3)^ z-transformed to a mean of 0 and a standard deviation of 1

^(4)^ dummy coded and centered with group ‘large’ (= starting with large template) being the reference category

**Table S3**. Results of separate models for each individual in Size test. (significant p-values are printed in bold)

| **subject** | **term** | **estimate** | **SE** | **𝜒^2^** | **df** | **p** |
| --- | --- | --- | --- | --- | --- | --- |
| Konrad | template size ^(1)^ | -2.863 | 0.495 | 13.131 | 1 | **< 0.001** |
|  | trial ^(2)^ | -0.326 | 0.253 | 1.375 | 1 | 0.241 |
| Figaro | template size ^(1)^ | -1.962 | 0.337 | 12.406 | 1 | **< 0.001** |
|  | trial ^(2)^ | -0.669 | 0.158 | 7.218 | 1 | **0.007** |
| Kiwi | template size ^(1)^ | -1.059 | 0.328 | 7.746 | 1 | **0.005** |
|  | trial ^(2)^ | -0.254 | 0.160 | 2.46 | 1 | 0.116 |
| Dolittle | template size ^(1)^ | -0.323 | 0.236 | 1.616 | 1 | 0.204 |
|  | trial ^(2)^ | 0.029 | 0.146 | 0.04 | 1 | 0.844 |
| Pipin | template size ^(1)^ | -0.55 | 0.34 | 2.574 | 1 | 0.109 |
|  | trial ^(2)^ | 0.087 | 0.17 | 0.259 | 1 | 0.611 |
| Fini | template size ^(1)^ | 1.237 | 0.744 | 1.731 | 1 | 0.188 |
|  | trial ^(2)^ | -0.111 | 0.388 | 0.081 | 1 | 0.776 |

^(1)^ dummy coded and centered with template size ‘large’ being the reference category

^(2)^ z-transformed to a mean of 0 and a standard deviation of 1

**Table S4**. Results of separate models for each condition in Size test (template size short or long; significant p-values are printed in bold)

| model | **term** | **estimate** | **SE** | **𝜒^2^** | **df** | **p** |
| --- | --- | --- | --- | --- | --- | --- |
| long template | intercept | 1.302 | 0.602 | ^(1)^ | ^(1)^ | ^(1)^ |
|  | trial ^(2)^ | -0.058 | 0.114 | 0.257 | 1 | 0.612 |
|  | group ^(3)^ | 0.051 | 0.849 | 0.004 | 1 | 0.952 |
| short template | intercept | -0.154 | 0.249 | ^(1)^ | ^(1)^ | ^(1)^ |
|  | trial ^(2)^ | -0.436 | 0.197 | 4.292 | 1 | **0.038** |
|  | group ^(3)^ | 0.971 | 0.366 | 5.126 | 1 | **0.024** |

^(1)^ not indicated because of having a very limited interpretation

^(2)^ z-transformed to a mean of 0 and a standard deviation of 1

^(3)^ dummy coded and centered with group ‘large first’ being the reference category

**Table S5**. Results of the full model of Shape test (estimates, together with standard errors, and confidence intervals, as well minimum and maximum of estimates obtained when excluding data points one at a time).

| term | Estimate | SE | t | p | lower Cl | upper Cl | min | max |
| --- | --- | --- | --- | --- | --- | --- | --- | --- |
| intercept | 13.510 | 0.550 |  | ^(1)^ | 12.424 | 14.597 | 13.336 | 13.669 |
| condition^(2)^ | 1.806 | 0.515 |  |  | 0.790 | 2.823 | 1.704 | 2.061 |
| indiv.^(3)^ | -0.300 | 0.476 |  |  | -1.241 | 0.641 | -0.451 | -0.179 |
| Session | -0.169 | 0.195 | -0.868 | 0.387 | -0.553 | 0.216 | -0.203 | -0.085 |
| Trial | -0.008 | 0.059 | -0.139 | 0.890 | -0.124 | 0.108 | -0.050 | 0.006 |
| condition:indiv. | 0.062 | 0.674 | 0.093 | 0.926 | -1.269 | 1.394 | -0.243 | 0.214 |

^(1)^ not indicated because of having a very limited interpretation

^(2)^ dummy coded with size test being the reference category

^(3)^ dummy coded with Kiwi being the reference category

**Table S6**. Results of the reduced model of Shape Test lacking the interaction between indivdual and condition (estimates, together with standard errors, and confidence intervals, as well minimum and maximum of estimates obtained when excluding data points one at a time).

| term | Estimate | SE | t | p | lower Cl | upper Cl |
| --- | --- | --- | --- | --- | --- | --- |
| intercept | 13.495 | 0.522 | 25.848 | ^(1^ | 12.463 | 14.526 |
| condition^(2)^ | 1.838 | 0.388 | 4.738 | <0.001 | 1.071 | 2.604 |
| indiv.^(3)^ | -0.269 | 0.336 | -0.800 | 0.425 | -0.932 | 0.395 |
| Session | -0.169 | 0.194 | -0.870 | 0.385 | -0.552 | 0.214 |
| Trial | -0.008 | 0.058 | -0.139 | 0.889 | -0.124 | 0.107 |

^(1)^ not indicated because of having a very limited interpretation

^(2)^ dummy coded with size test being the reference category

^(3)^ dummy coded with Kiwi being the reference category

**References**

Auersperg AMI, Borasinski S, Laumer I, Kacelnik A. 2016 Goffin’s cockatoos make the same tool type from different materials. *Biol. Lett*. **12**: 20160689.

<http://dx.doi.org/10.1098/rsbl.2016.0689>
